# The Book World of Medicine and Science

**Published:** 1894-07-28

**Authors:** 


### The Book World of Medicine and Science.

### BOOK REVIEWS.

Travels in Search of New Trade Products. By Arthur Robottom. (London: Jarrold and Sons.)

This little work, besides being a record of personal adventure and incident, is written with the object of collating in a readable form those particulars which relate to the industrial development of the country. From his immense experience of commercial matters, and from his untiring enterprise in seeking after new commodities and raw materials, a work from Mr. Robottom's pen cannot fail to be of value to those interested in commercial development and trade industries. An idea of the book may be gained from the following paragraph, with which the author introduces his work to his readers: "One year I have been scouring the continent of Europe for stray goods for which no commercial use has been found; another year I have been in Asia Minor, North, South, or Central America, or the West Indies, exploring for new raw materials. . . . of course I have been much of a 'rolling stone,' and have consequently not 'gathered moss' to such an extent as I might have done perhaps had I been of a less adventurous disposition. Still, I have had my successes, and have made quite as many fortunes as I have lost." Perhaps one of the most interesting enterprises in which Robottom was engaged was that which concerned the introduction of borax to the public at a cheap rate. Thus he succeeded in proving at an exhibition at the Agricultural Hall that a packet of borax could be sold for a penny and a profit secured. The Birmingham Patent Borax Company was formed with this object, and such success has attended the enterprise that at the present time it affords employment for 900 hands. Mr. Robottom is at present engaged in treating boracic acid in a similar manner. Should this attempt succeed, and the public thus enabled to buy in penny packets this most valuable antiseptic, the result will be a valuable one. In our hospitals the value of boracic acid as a non-poisonous, almost tasteless, and cleanly antiseptic has been fully recognised. The public, however, have still to learn that as an internal antiseptic, or as an external application to wounds, whether in the form of powder or in solution, boracic acid has at present no formidable rival.

The New Guide to Bristol and Clifton and the Bristol Channel Circuit. Edited by James Baker, F.R.G.S., with articles by Professor Lloydd Morgan, J. White, F.R.S., C. Wilson, F.G.S., E. Norris Matthews, Thomas Evans, &c. Illustrated, pp. 312. (Clifton and London: J. Baker and Sons.)

This handy little guide to Bristol and its environs is written for presentation to the members of the British Medical Association attending the annual meeting in the coming week. The first few pages are occupied by the official programme of this meeting. A little further on are very complete lists of the chief hotels, restaurants, clubs, hospitals, art galleries, &c., which cannot fail to be useful to strangers. The editor's power of graphically describing scenery and places of interest in a pleasant and natural style has rendered him peculiarly well adapted to a work of this description. Professor Lloydd Morgan and the other contributors deal with the main points of interest in the physical features of the geology and botany of the beautiful gorge of the Avon, and these special chapters render the book as complete as it is possible to make a guide of such a small and convenient size.

Prescription Books. (Bristol: T. Wright and Co.)

The above firm have forwarded us a sample of their prescription book. It is a neatly-bound book, with gilt edges and perforated leaves. The size is such as can be conveniently carried in the pocket, and the book is well got up and all that can be desired as far as convenience and appearance is concerned. A somewhat similar book for duplicate prescriptions is issued by the same firm.

